# Development of a multiplex droplet digital PCR method for detection and monitoring of *Mycobacterium tuberculosis* and drug-resistant tuberculosis

**DOI:** 10.1186/s12941-024-00687-2

**Published:** 2024-04-05

**Authors:** Yu Jeong Choi, Yoonjung Kim, Hye Jung Park, Dokyun Kim, Hyukmin Lee, Young Ah Kim, Kyung-A Lee

**Affiliations:** 1 Department of Laboratory Medicine, Gangnam Severance Hospital, Yonsei University College of Medicine, 211, Eonju-ro, Gangnam-gu, Seoul, 06273 Korea; 2 Department of Internal Medicine, Yonsei University College of Medicine, Seoul, Korea; 3 Department of Laboratory Medicine, National Health Insurance Service Ilsan Hospital, Goyang, Korea

**Keywords:** *Mycobacterium tuberculosis*, Multidrug-resistant tuberculosis, Multiplex ddPCR

## Abstract

**Background:**

The prevalence of multidrug-resistant tuberculosis (MDR-TB) among Korean tuberculosis patients is about 4.1%, which is higher than the OECD average of 2.6%. Inadequate drug use and poor patient compliance increase MDR-TB prevalence through selective pressure. Therefore, prompt detection of drug resistance in tuberculosis patients at the time of diagnosis and quantitative monitoring of these resistant strains during treatment are crucial.

**Methods:**

A multiplex droplet digital PCR (ddPCR) assay was developed and assessed using DNA material of nine *Mycobacterium tuberculosis* strains with known mutation status that were purchased from the Korean National Tuberculosis Association. We collected a total of 18 MDR-TB residual samples referred for PCR analysis. Total DNA was extracted from the samples and subjected to the quadruplex ddPCR assay. Their results were compared to those of known resistance phenotypes.

**Results:**

The analytical sensitivity and specificity of the multiplex ddPCR assay for detecting INH, RIF, EMB, FQ, and SM resistance-causing mutations ranged from 71.43 to 100% and 94.12–100%, respectively. Follow-up sample results showed that the quadruplex ddPCR assay was sensitive enough to detect *IS6110* and other mutations even after onset of treatment.

**Conclusions:**

We developed a sensitive and accurate multiplex ddPCR assay that can detect the presence of tuberculosis quantitatively and resistance-conveying mutations concurrently. This tool could aid clinicians in the diagnosis and treatment monitoring of tuberculosis.

**Supplementary Information:**

The online version contains supplementary material available at 10.1186/s12941-024-00687-2.

## Background

Pulmonary tuberculosis is a bacterial infection caused by *Mycobacterium tuberculosis* (MTB). It is highly contagious; about 30% of close contacts with TB patients are infected, and about 10% of these infected individuals develop pulmonary TB. According to the World Health Organization (WHO) Global Tuberculosis Report 2023, 7.5 million people were newly diagnosed with TB and 1.3 million died [1]. Korea has the highest prevalence of TB among Organization for Economic Co-operation and Development (OECD) countries, with around 3 million newly diagnosed TB patients and 2,000 deaths from TB every year, as documented by the Korean Centers for Disease Control and Prevention (KCDC) in their annual report [2]. Moreover, the prevalence of multi-drug resistant tuberculosis (MDR-TB) among Korean TB patients is about 4.1%, which is higher than the OECD average of 2.6% [3]. Inadequate drug use and/or poor patient compliance increase MDR-TB prevalence through selective pressure [4, 5]. Therefore, prompt detection of drug resistance in TB patients at the time of diagnosis and quantitative monitoring of these resistant strains during treatment are crucial.

The gold standard method to diagnose TB and detect resistance is through bacterial culture [2]. However, due to the extended 6 to 8 weeks required to obtain results with this method, molecular tests are also widely used for rapid diagnosis and detection of drug resistance. Real-time polymerase chain reaction (RT-PCR) is a widely used method for timely identification of MTB and its resistance patterns. The Xpert MTB/RIF assay (Cepheid, Sunnyvale, CA, USA) is an automated semi-quantitative, nested, real-time PCR assay for rapid, simultaneous detection of MTB and rifampicin (RIF) resistance. With respect to RIF resistance detection, the Xpert MTB/RIF Ultra assay exhibits high sensitivity (92.7–95%) and specificity (98–99%) [6, 7]. However, this RT-PCR assay is not suitable for high-throughput analysis, which is crucial for laboratories handling large sample sizes. Moreover, precise quantification is not achievable, limiting the assay usefulness in monitoring patient compliance and heteroresistance.

Digital droplet PCR (ddPCR) is a promising tool for detecting resistant strains and monitoring therapeutic response with better sensitivity than conventional RT-PCR [8–10]. ddPCR can accurately measure the absolute nucleic acid count of single template molecules without the need for standard curves, is less susceptible to interference from PCR inhibitors, and can generate up to 20,000 droplets per sample, allowing a very low limit of detection (0.005%) [8–10]. Based on these advantages, we speculated that ddPCR could be used to develop a new, highly sensitive method for rapid and accurate diagnosis of TB that has the ability to detect multiple drug resistance variants simultaneously and quantitatively.

To achieve this goal, we developed a ddPCR-based multiplex panel that can simultaneously target eight genes, encompassing 11 distinct targets (*rpoB* S450L, H445Y, D435V; *inhA* C(-15)T; *katG* S315T; *embB* M306V, M306I; *gyrA* D94G; *rrs* A1401G; *rpsL* K43R; *IS6110*). This panel covers the most prevalent resistance-conveying variants of each representative resistance gene as a proof-of-concept prototype.

As mentioned earlier, rapid and accurate TB diagnosis, especially prompt recognition of drug resistance and compliance monitoring, is crucial for proper management of TB. Therefore, we aimed to demonstrate ddPCR-based multiplex panel assay as a promising tool for quantitative measurement of TB and its drug resistance profile.

## Methods

### Primers and probes

The primer-probe set used to target *IS6110* was reported previously [11]. Before designing the primer-probe sets targeting the resistance genes *rpoB*, *katG*, *inhA*, *embB*, *gyrA*, *rpsL*, and *rrs*, we conducted a comprehensive literature review to identify the prevalent mutations associated with drug resistance for each gene (Supplementary Table 1). Using those as our targets, primers and probes were designed with the Primer and Probes Design Tool offered by GenScript (Piscataway, NJ, USA). *InhA*, *rpoB*, *rpsL*, and *gyrA* probes were labeled with the fluorophore FAM, while *katG*, *rrs*, and *IS6110* probes were labeled with VIC. *EmbB* probes were labeled with HEX. The sequences, concentrations, and attached dyes of the two multiplex panels for ddPCR are summarized in Supplementary Table 2. A primer-probe set for pyrazinamide was not included in the assay since the reported mutations in *pncA* were dispersed throughout the sequence of the gene.

### Specimens

We received DNA material of nine MTB strains with known mutation from the Korean National Tuberculosis Association, and these were used for assay development and evaluation (Supplementary Table 3). These strains had mutations in *rpoB*, *katG*, *inhA*, *embB*, *gyrA*, *rpsL*, or *rrs* genes, as well as two wild type strains. To evaluate the performance of the quadruplex ddPCR assay, we additionally received eight cultured MTB strains with known drug sensitivity phenotypes based on a drug susceptibility test (DST). A total of 18 residual sputum samples from 16 drug-resistant TB patients was collected between 2019 and 2022 at two tertiary hospitals, including initial and/or follow-up samples from drug-resistant TB patients who initially tested culture-positive. The demographic characteristics of the 18 samples obtained from 16 patients with mono-resistance or multidrug resistance (MDR) are summarized in Supplementary Table 4. The median age of the patients was 66.5 years, and there was a male predominance. Regarding sample type, the majority (88.9%) were sputum samples, while the rest (11.1%) were bronchial lavage samples. In cases of resistance phenotype, 50% of the patients harbored strains that were resistant to isoniazid, and 43.8% were infected with rifampicin-resistant strains. These samples were used to validate quadruplex ddPCR assays, assess the usefulness of the multiplex panel assay in monitoring TB treatment, and compare its results with that of the Xpert MTB/RIF assay.

Research involving human specimens complied with all relevant national regulations, institutional policies, and the tenets of the Helsinki Declaration (as revised in 2013). The study was approved by the Institutional Review Board of Severance Hospital, Seoul, Korea (IRB no. 3-2020-0455). The requirement for informed consent was waived.

### DNA extraction

For total DNA analysis, 100 µL aliquots of the clinical specimens were re-suspended in DNA extraction buffer. Total DNA was extracted using a QIAamp DNA mini kit (QIAGEN, Hilden, Germany) as stated in the manufacturer’s instructions.

### ddPCR

**Fig. 1:**
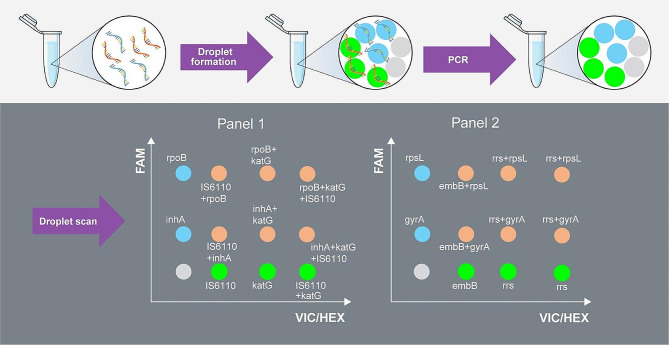
The sample mix undergoes droplet formation so that the DNA strands are compartmentalized into separate droplets. Then, they are amplified via PCR, and the amplified product reacts with the fluorescence-labeled probe. Each droplet is scanned and plotted in its corresponding coordinates. The x-axis represents signal detected from Channel 2 (VIC/HEX), while the y-axis represents signal detected from Channel 1 (FAM)

Digital PCR reactions were performed with a QX200 Droplet Digital PCR System (Bio-Rad Laboratories, Hercules, CA, USA). The ddPCR reaction mixture was composed of a 10 µL dPCR Probe Supermix (Bio-Rad, CA, US), 1800/900/450 nM primers per target, 500/250/125 nM probe per target, and 1 µL of sample. Ultrapure DNase- free water was added to the reaction mixture to a final volume of 20 µL. Then, micro-droplets were generated from the mixture via QX200 Droplet Generator (Bio-Rad). Results were analyzed with QuantaSoft Analysis Pro software (Bio-Rad Laboratories). This provided the number of positive and negative droplets, quantification of *IS6110* of MTB, and resistance-related genes expressed as copies/uL of ddPCR reaction. At least two positive droplets were required for a ddPCR assay reaction to be interpreted as positive. The procedure of multiplex ddPCR is summarized in Fig. 1, and the actual 2D plots of our assay are shown in Supplementary Fig. 1. The amplitude multiplex ddPCR method was used to distinguish targets/target sets.

### Limits of detection

We designed two multiplex ddPCR assays to detect mutations on *rpoB*, *katG*, *inhA*, *embB*, *gyrA*, *rpsL*, and *rrs*, as well as the *IS6110* gene specific to the *Mycobacterium tuberculosis* complex. *InhA*, *rpoB*, *katG*, and *IS6110* were combined as panel 1; while *embB*, *gyrA*, *rpsL*, and *rrs* were grouped as panel 2. Twenty TB PCR-negative sputum samples were tested to determine the limit of blank (LoB). The assay performance evaluation including sensitivity and specificity was validated as recommended [12]. Four DNA concentrations (50, 10, 5, and 2 copies/µL) using g-block (IDT, Redwood City, CA, USA) were prepared for each corresponding probe to determine the limit of detection (LoD). The two higher concentrations (50, 10 copies/µL) were tested four times, whereas eight replicates were tested for the lower two (5, 2 copies/µL).

### Statistical analysis

Microsoft Excel 2013 (Seattle, WA, USA), Analyse-it for Microsoft Excel Method Evaluation Edition version 5.40.2 (Analyse-it Software, Ltd., Leeds, UK), and SPSS Statistics v.23 (SPSS, Inc., Chicago, IL, USA) were used for statistical analysis. Porbit analysis was conducted to obtain LoD values.

## Results

### Determination of limit of blank (LoB) and limit of detection (LoD)

The LoB for the targeted genes *IS6110*, *katG*, *inhA*, *rpoB*, *embB*, *rrs*, *gyrA*, and *rpsL* were 1, 0, 1.5, 0.5, 1.5, 0.5, 1.5, and 0 copies per reaction, respectively. To ensure reliable detection, a cut-off of 2 positive droplets was implemented. The LoD was determined using probit analysis for each probe, yielding the following values: *rpoB*_435, 3.09 copies/µL; *rpoB*_445, 2.97 copies/µL; *rpoB*_450, 2.83 copies/µL; *inhA*, 2.97 copies/µL; *IS6110*, 2.97 copies/µL; *katG*, 2.97 copies/µL; *gyrA*, 3.23 copies/µL; *rpsL*, 3.11 copies/µL; *embB_I*, 2.66 copies/µL; *embB_V*, 2.86 copies/µL; and *rrs*, 2.67 copies/µL.

### Analytical performance evaluation of the quadruplex ddPCR assay

The sensitivity and specificity of the quadruplex ddPCR assay for detecting RIF resistance-causing mutations were both 100%. The assay sensitivity and specificity ranged from 71.43 to 100% and 94.12–100%, respectively, for isoniazid (INH), ethambutol (EMB), fluoroquinolone (FQ), and streptomycin (SM) resistance-causing mutations. The exact values and 95% confidence intervals (CIs) for each target are shown in Table 1.

The results obtained from quadruplex ddPCR assays of eight MTB strains with known drug sensitivity phenotypes are summarized in Table 2. The analytical sensitivity and specificity of the multiplex ddPCR assay regarding resistance phenotype obtained by DST were 80.00% (95% CI: 56.34–94.27) and 91.67% (95% CI: 77.53–98.25), respectively. The resistance phenotype of pyrazinamide (PZA) was excluded from the comparison of phenotype and genotype since the quadruplex ddPCR assay did not include probes for *pncA*. The discrepancies (false positives as well as false negatives) observed between the resistance phenotypes and resistance gene mutation statuses were as follows. T-3 exhibited INH resistance, but no mutations were detected in either *katG* or *inhA*. T-2 and T-6 were resistant to EMB, while T-3 was sensitive. However, an *embB* mutation was detected exclusively in T-3. All the strains tested were sensitive to FQ (ofloxacin and moxifloxacin) phenotypically, but mutation on *gyrA* was detected in T-5 and T-7. Last, T-2 was resistant to SM, but no mutations were detected on either *rpsL* or *rrs*. Sanger sequencing of these discrepant sites confirmed that the ddPCR results were correct sequence-wise.

### Validation of quadruplex ddPCR assay using drug-resistant patient samples

The quadruplex ddPCR results were assessed with eight lower respiratory tract specimens (sputum and bronchial washing fluid) collected simultaneously to culture-positive specimens. The multiplex ddPCR results of the initial patient samples are summarized in Table 3. The quadruplex ddPCR assay successfully quantified the *IS6110* target in all samples (100%, *n* = 8/8). Furthermore, overall molecular and phenotypic resistance patterns correlated with ddPCR results. Of the seven specimens with phenotypic resistance pattern results, five were fully concordant (71.4%), and two were partially concordant (28.6%). Sample G-1 was resistant to RIF, but no *rpoB* mutation was detected on either ddPCR or Xpert RIF assay. Sample S-2 contained EMB-resistant strains that tested negative for *embB* mutation by ddPCR. In sample S-8, drug sensitivity testing failed due to low bacterial culturability, while the Xpert RIF assay was positive. However, ddPCR did not detect the *rpoB* mutation. Sanger sequencing of the rifampicin resistance-determining region (RRDR) revealed that it harbored a mutation on codon 432 not covered by the quadruplex ddPCR panel, explaining the discrepancy between ddPCR and Xpert RIF results.

### Use of quadruplex ddPCR assay in the follow-up setting

Residual sputum samples were collected at various time points during the treatment course of patients with initial culture positivity, and multiplex ddPCR was performed in the follow-up of MDR-TB patients. A total of 10 samples was included, consisting of four culture-positive samples, four negative samples, and two undetermined samples. The multiplex ddPCR results of the follow-up patient samples are summarized in Table 4. The quadruplex ddPCR assay was able to simultaneously quantify *IS6110* and drug-resistant mutations in the same reaction in culture positive samples (S-1, S-3 and S-9) except the S-13 sample. The discrepancies observed between the resistance phenotypes and the results of the ddPCR assay were as follows: S-3 showed phenotypic resistance to INH, RIF, and EMB; and ddPCR results showed corresponding findings but with an additional *rpsL* mutation. In S-9, no drug-resistance mutations were observed in the quadruplex ddPCR assay based on the phenotype of the initial sample, which may reflect treatment-induced changes given the concurrent values of copies of the *IS6110* gene (6.23 copies/reaction) and *inhA* mutation (2.08 copies/reaction). The Xpert RIF assay was conducted on a total of nine specimens, and six of them showed the same results (66.7%, *n* = 6/9) as those of the quadruplex ddPCR assay. Disagreements between the ddPCR result and the Xpert RIF assay results were found in S-9, S-13, and S-14. The *IS6110* copies per reaction ranged from 6.23 to 11.38, and the probe B region of the Xpert RIF assay (detected in S-14) was not covered in ddPCR.

## Discussion

Overall, genotypic results of multiplex ddPCR assays correlated well with the resistance phenotype. However, some exceptions were noted that need explaining. Regarding discrepancies found on multiplex ddPCR results of eight MTB strains, Sanger sequencing confirmed that the ddPCR results were accurate at the sequence level. According to prior research, the most frequent forms of discrepancy were genotypic susceptibility and phenotypic resistance to isoniazid, as with T-3 [13]. Those authors speculated that rare mutations such as those in *kasA* or *msbA* or, more commonly, *ahpC* could be the reason for such disparity. Discrepant results for *embB* were observed for three of eight strains: two were phenotypically resistant but genetically susceptible (T-2, T-6), while one was phenotypically susceptible but genetically resistant (T-3). Discordance in both ways has been reported by Ahmad et al., where the agreement between phenotypic resistance and genotypic resistance was lowest for ethambutol (only 76% compared to 96% and 97% for rifampicin and isoniazid, respectively) [14]. This report is also in line with our finding that the sensitivity for EMB was the lowest at 71.43% (Table 2). *GyrA* mutation was detected in two fluoroquinolone-sensitive strains (T-5, T-7), though at very low concentrations: 6.32 and 3.30 copies per reaction. Heteroresistance is a possible mechanism to explain this situation [15]. If this is the case, detection of mutation at such low levels can prevent the development of drug resistance that may arise through selective pressure. As for the *rpsL* mutation that was not detected in a strain resistant to streptomycin (T-2), mutation at another site could be the reason for this discrepancy.

Regarding discrepancies found in the quadruplex ddPCR results of 18 patient samples, Sanger sequencing could not be performed due to lack of remnant samples. However, the observed discrepancies were like those described above. In the initial samples (Table 3), the lack of *rpoB* mutation despite phenotypic RIF resistance in G-1 was confirmed by Xpert RIF (Supplementary Table 5). Nevertheless, since MTB strains harboring mutations such as I419F cannot be detected by GeneXpert and are low level resistance mutations, such may have been the case for G-1. The S-2 phenotypic EMB resistance despite lack of mutation in *embB* may be due to a mutation not covered by our panel. G-6.1 and S-7 were pan-susceptible, but sequencing conducted according to the clinician’s instructions revealed a mutation in the *inhA* gene that was confirmed through ddPCR. G-5.1 was also pan-susceptible, but sequencing revealed mutation in *inhA* not detected by ddPCR, probably because it was at a site not covered by our probe. ddPCR detected a mutation in *katG*, indicating that this strain had mutations in both *inhA* and *katG* genes. Three culture negative samples tested positive for *IS6110* (G-6.2, S-7, S-14), but they were all collected after onset of treatment (Table 4). Two ethambutol-resistant strains tested negative for mutation (S-2, S-9). *RpsL* mutation was detected on one streptomycin-sensitive sample (S-3), while one streptomycin-resistant sample tested negative for mutation (S-9). No mutation on *rpoB* was observed in S-9, but Xpert RIF was positive for mutation. The reason for the S-9 discordance between the ddPCR result and the Xpert result can be found in its *IS6110* level of 6.23 copies/reaction. MTB usually has multiple copies of *IS6110*, up to 25 copies per genome [16]. Moreover, the *M. tuberculosis* Beijing/W lineage, which is prevalent in East Asia [17], has an exceptionally high copy number of *IS6110*. When considering these facts, the copy number of *rpoB* is predicted to be less than 1 copy per reaction, which explains why the mutation was not detected.

One unique feature of the patient sample results was that the culture results could be compared whether or not *IS6110* was detected by the ddPCR assay. The discrepancy between them went both ways; there were samples that were culture negative but *IS6110* was detected and samples that were culture positive but *IS6110* was not detected. The detection of *IS6110* by ddPCR in samples that were negative by culture can be explained by the fact that culture only identifies live bacteria, while ddPCR can detect both viable and non-viable MTB. Nevertheless, it cannot be ruled out that conventional diagnostic tests may yield negative results due to the relatively low abundance of MTB in the sample. The latter (S-13) situation, where the culture is positive but molecular method fails to detect MTB, is less intuitive, yet they have been reported [18]. If the specimen has a low bacterial load because the amount of specimen needed for ddPCR is so small, the portion of sputum used for the PCR may not contain any MTB by chance. It is also possible that the strain might have been *IS6110*-negative. In fact, such strains have been reported and traced to southeast Asia [19], as well as in certain regions in Korea [20].

Sensitivity of the ddPCR assay relative to pDST for isoniazid was 93.33% (95% CI: 68.05–99.83) and specificity was 100.00% (95% CI: 84.56–100.00), which were comparable to those of Xpert MTB/XDR assays of 98.3% (95% CI: 95.8–99.3) and 95.0% (95% CI: 73.1–99.7), respectively [21]. In the case of rifampicin, we observed a sensitivity and specificity of 100.00% (95% CI: 78.20–100.00) and 100.00% (95% CI: 84.56–100.00), superior to the previously reported sensitivity of 81.0% (95% CI: 74.9–86.2) and specificity of 98.7% (95% CI: 93.0–100) for rifampicin using Xpert [6]. When the benefits of multiplex ddPCR are considered, our new method could prove to be more useful, especially in labs dealing with large sample quantities.

The fact that five of 16 strains with suspected resistance had unknown phenotypes was also noteworthy (Supplementary Table 4). This meant that phenotypic drug resistance could not be determined for 31.25% of TB patients. They were either too heavily contaminated by other bacteria, nothing grew in the culture test, or the culturability of MTB was too weak to carry out a drug sensitivity test despite a positive culture test. Since molecular methods can overcome these obstacles, multiplex ddPCR could aid in predicting resistance patterns and guide clinicians as to which drug combination to use in such cases.

ddPCR results of follow-up samples demonstrated that the quadruplex ddPCR assay was sensitive in detecting *IS6110* and other mutations even after onset of treatment. Based on these findings, it is apparent that our multiplex ddPCR assay holds promise for facilitating not only the initial diagnostic phase but also treatment monitoring. The consistent decline or absence thereof in specific markers provides clinicians with valuable insights into patient drug adherence, potential emergence of heteroresistance, and other pertinent factors.

Some of the limitations of our study are described here. First, the number of samples collected was too small. Unfortunately, our sample collection period overlapped with the COVID-19 pandemic, during which the number of TB patients dropped either because fewer were infected due to the wearing of masks and thorough washing of hands or access to TB diagnostic services decreased the number of cases diagnosed regardless of the actual prevalence [22]. Reflecting such factors, the prevalence of MDR-TB, which used to be around 4% before the pandemic [3] was reported to have decreased to 1.57% and 1.62% on the years 2020 and 2021 [23]. Another limitation was lack of an internal control due to the restricted number of detection channels. Also, since synthetic DNA was used when assessing LoD, interference arising from Mtb DNA might have been overlooked. Last, mutations in regions not included in our assay could have been missed. Sanger sequencing of the RRDR region of S-8 revealed a mutation on codon 432 not covered by our panel, explaining the discrepancy between ddPCR and Xpert RIF results (Supplementary Table 5). Further study using a larger sample of drug-resistant TB samples with known resistance phenotype as well as adding more probes for mutation points that are prone to occur but not included in this assay could aid in assessing the utility of multiplex ddPCR more accurately.

## Conclusion

In conclusion, we developed a sensitive and accurate multiplex ddPCR assay that can detect the presence of TB as well as resistance-conveying mutations concurrently in a quantitative way. This tool could aid clinicians in the diagnosis and treatment monitoring of TB.

**Table 1:** Analytical sensitivity and specificity of the quadruplex ddPCR assay evaluated using nine DNA samples, eight cultured MTB samples with known drug resistance phenotype, and 20 culture-negative samples

Drug	TP	FP	FN	TN	Sensitivity (%)^a^	95% CI	Specificity (%)^a^	95% CI
INH	14	0	1	22	93.33	68.05–99.83	100.00	84.56–100.00
RIF	15	0	0	22	100.00	78.20–100.00	100.00	84.56–100.00
EMB	5	1	2	29	71.43	29.04–96.33	96.67	82.78–99.92
FQ	3	2	0	32	100.00	29.24–100.00	94.12	80.32–99.28
SM	7	0	1	29	87.50	47.35–99.68	100.00	88.06–100.00

TP, true positive; FP, false positive; FN, false negative; TN, true negative

^a^ The nine DNA sample result was compared with the mutation profiles provided to us by the Korean National Tuberculosis Association while the eight cultured MTB sample result was compared with the phenotypic resistance result

**Table 2:** Resistance phenotype and ddPCR results of eight live MTB strains

Sample No.	Phenotypic resistance (Lowenstein-Jensen medium)	Molecular resistance (ddPCR)	Discrepancies between phenotype and genotype	Results of confirmation using Sanger sequencing
T-1	INH, RIF	*katG, rpoB*		
T-2	INH, RIF, EMB, SM	*katG, rpoB*	EMB; SM	*embB* (M306V, M306I) and *rpsL* (K43R): ND
T-3	INH, RIF, PZA	*rpoB, embB*	INH; *embB*	*inhA* (C(-15)T) and *katG* (S315T): ND *embB* (M306I): D
T-4	INH, RIF	*inhA, rpoB*		
T-5	INH, RIF, SM	*katG, rpoB, gyrA, rpsL*	*gyrA*	*gyrA* (D94G): D
T-6	INH, RIF, EMB	*katG, rpoB*	EMB	*embB* (M306V, M306I): ND
T-7	INH, RIF, EMB, SM	*katG, rpoB, embB, gyrA, rpsL*	*gyrA*	*gyrA* (D94G): D
T-8	INH, RIF, PZA	*katG, rpoB*		

ND, not detected; D, detected

**Table 3:** Quadruplex ddPCR results of initial MDR-TB patient samples

Sample No.	Sample type	AFB smear	Molecular resistance (sequencing)	Phenotypic resistance	Culture	ddPCR (copies/reaction)	Xpert		Probe
							MTB	RIF	
G-1	Sputum	-	INH, RIF	INH, RIF, PZA	Positive	*IS6110* (32.41)	Negative	Negative	-
						*inhA* (36.02)			
G-3	Sputum	-	INH, RIF	INH, RIF, PZA	Positive	*IS6110* (45.29)	Positive	Positive	probe E
						*inhA* (9.93)			
						*rpoB* (2.21)			
G-4	Sputum	-	INH	INH	Positive	*IS6110* (1493.43)	Positive	Negative	-
						*katG* (66.58)			
						*inhA* (75.95)			
G-5.1	Bronchial lavage	2+	INH	Pan-susceptible	Positive	*IS6110* (63.65)	Positive	Negative	-
						*katG* (7.67)			
G-6.1	Sputum	-	INH	Pan-susceptible	Positive	*IS6110* (946.90)	NT	NT	NT
						*inhA* (44.35)			
G-7	Sputum	-	INH	Pan-susceptible	Positive	*IS6110* (160733.52)	NT	NT	NT
						*inhA* (6236.44)			
S-2	Sputum	3+	INH, RIF	INH, RIF, EMB	Positive	*IS6110* (11.00)	Positive	Positive	probe E
						*inhA* (3.30)			
						*rpoB* (2.20)			
S-8^a^	Sputum	2+	-	-	Positive	*IS6110* (1302.16)	Positive	Positive	probe B
						*katG* (67.68)			

^**a**^ Drug sensitivity test (DST) failed due to low bacterial culturability, Xpert RIF test was positive

**Table 4:** Quadruplex ddPCR results of follow-up MDR-TB patient samples

Sample No.	Sample type	Tx Hx	AFB smear	Molecular resistance (sequencing)	Phenotypic resistance	Culture	ddPCR (copies/reaction)	Xpert		Probe
								MTB	RIF	
G-2^a^	Sputum	17 months of Tx	-	INH, RIF	INH, RIF, EMB, PZA	Negative	*-*	Negative	Negative	-
G-5.2	Sputum	2 days of HERZ	2+	NT	NT	NT	*IS6110* (2.33)	Negative	Negative	-
G-6.2	Bronchial lavage	3 months of HERZ	-	NT	NT	Negative	*IS6110* (268.66)	NT	NT	NT
							*inhA* (15.46)			
S-1	Sputum	3 weeks of HERZ	-	NT	NT	Positive	*IS6110* (162.28)	Positive	Positive	probe E
							*inhA* (15.20)			
							*rpoB* (13.03)			
S-3	Sputum	2 months of HERZ, 3 weeks of Z + H + Q	1+	INH, RIF	INH, RIF, EMB	Positive	*IS6110* (195.64)	Positive	Positive	probe E
							*inhA* (20.81)			
							*rpoB* (7.66)			
							*embB* (14.54)			
							*rpsL* (3.11)			
S-4^b^	Sputum	1year of Tx (on and off)	2+	NT	NT	Positive	*IS6110* (124.50)	Positive	Positive	probe E
							*inhA* (4.36)			
							*rpoB* (10.89)			
S-7	Sputum	4 months of HERZ	-	NT	NT	Negative	*IS6110* (9.93)	Positive	Positive	probe A
							*inhA* (3.31)			
							*rpoB* (1.14)			
S-9 ^a^	Sputum	3 months of HERZ	-	RIF	INH, RIF, EMB, PZA, FQ, SM	NT	*IS6110* (6.23)	Positive	Positive	probe E
							*inhA* (2.08)			
S-13 ^a^	Sputum	2 months of HERZ	-	INH, RIF	INH, RIF, EMB, SM	Positive	*-*	Positive	Positive	probe E
S-14	Sputum	1 months of HERZ	-	NT	NT	Negative	*IS6110* (11.38)	Positive	Positive	probe B

H, isoniazid; R, rifampin; E, ethambutol; Z, pyrazinamide; Q, fluoroquinolone; NT, not tested; -, not detected

^a^ pDST and Xpert RIF test from initial positive strain prior to treatment

^**b**^ Drug sensitivity test (DST) failed due to low bacterial culturability

### Electronic supplementary material

Below is the link to the electronic supplementary material.

Supplementary Material 1

Supplementary Material 2

## Data Availability

All data generated or analysed during this study are included in this published article and its supplementary information files.

## Abbreviations

CI: confidence interval
ddPCR: droplet digital polymerase chain reaction
DST: drug susceptibility test
EMB: ethambutol
FQ: fluoroquinolone
INH: isoniazid
LoB: limit of blank
LoD: limit of detection
MDR-TB: multi-drug resistant tuberculosis
MTB: *Mycobacterium tuberculosis*
OECD: Organization for Economic Cooperation and Development
RIF: rifampicin
RT-PCR: Real-time polymerase chain reaction
SM: streptomycin
WHO: World Health Organization
