# Supplementary figures and images for: Development of a multiplex droplet digital PCR method for detection and monitoring of Mycobacterium tuberculosis and drug-resistant tuberculosis
Source: Ann Clin Microbiol Antimicrob. 2024 Apr 5;23:29. doi: 10.1186/s12941-024-00687-2 (PMC10998390; doi:10.1186/s12941-024-00687-2)

Supplementary Figure 1. 2D plots of panel 1 (A) and panel 2 (B) of our ddPCR assay.


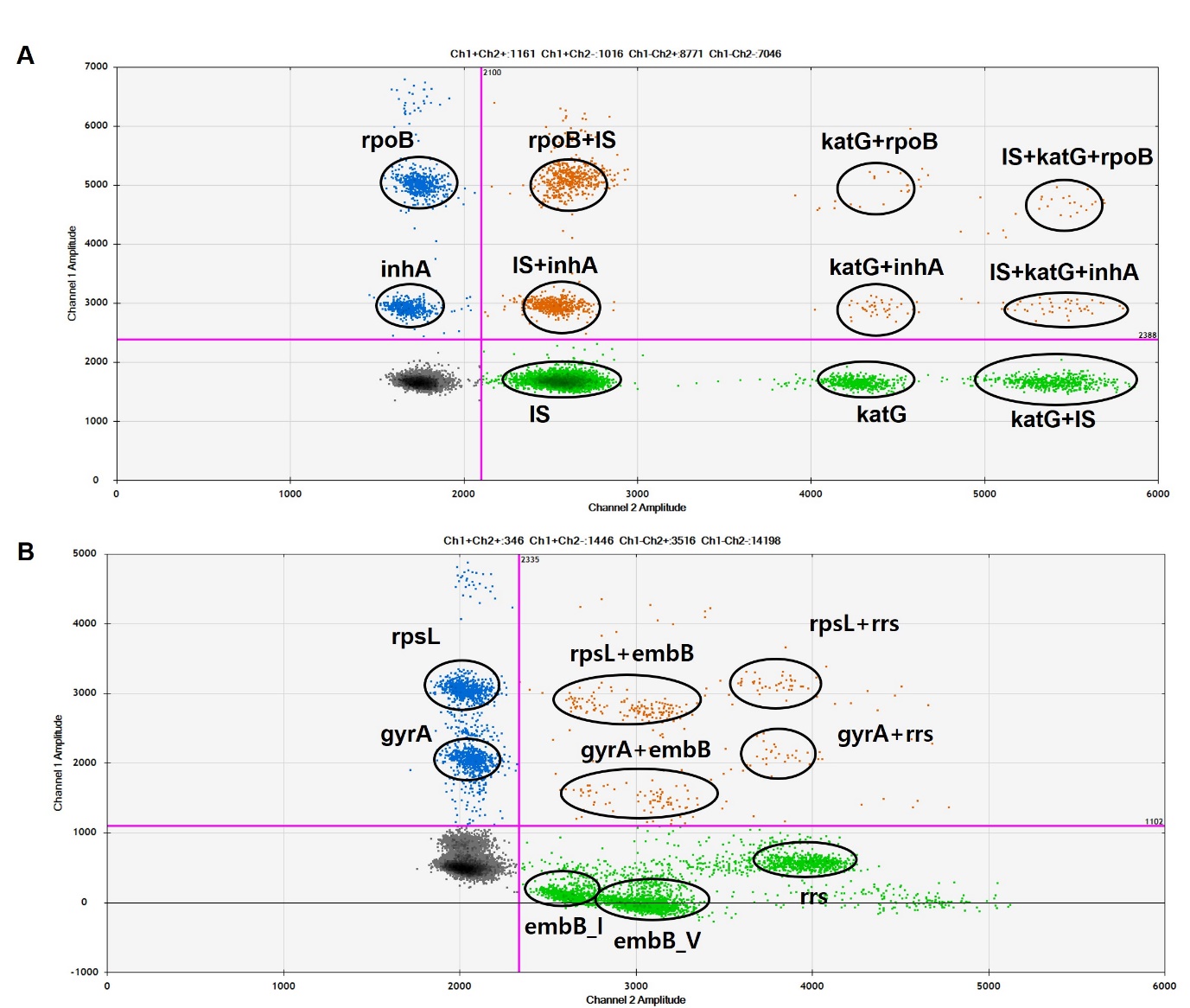

Supplement: Supplementary file 1 — Supplementary Material 1 [file 12941_2024_687_MOESM1_ESM.docx]
