# Supplementary material for: Development of a multiplex droplet digital PCR method for detection and monitoring of Mycobacterium tuberculosis and drug-resistant tuberculosis
Source: Ann Clin Microbiol Antimicrob. 2024 Apr 5;23:29. doi: 10.1186/s12941-024-00687-2 (PMC10998390; doi:10.1186/s12941-024-00687-2)
Supplement: Supplementary file 2 — Supplementary Material 2 [file 12941_2024_687_MOESM2_ESM.docx]

Supplementary Table 1. Summary of resistance mutations reported in prior studies

| Study (yr) | RIF | INH | | EMB | PZA | FQ | SM | KM | AMK |
| --- | --- | --- | --- | --- | --- | --- | --- | --- | --- |
|  | *rpoB* | *katG* | *inhA* | *embB* | *pncA* | *gyrA* | *rpsL* | *rrs* | *rrs* |
| Jnawali *et al.* (2013) (23) | **S531L(44.79)** | **S315T(30.2)** | **C(-15)T (21.4)** | **M306V(23.4)** | L159R(8.3) | **D94G(32.8)** | **K43R(12.5)** | **A1401G(7.3)** | **A1401G(7.3)** |
|  | D516V(8.3) |  |  | **M306I(17.2)** | T135P(3.6) | A90V(12.0) | K88R(4.7) |  |  |
|  | **D516Y(13.54)** |  |  | Q497R(7.8) | D12A(2.6) | S91P(10.0) |  |  |  |
|  | **H526Y(9.4)** |  |  | Q497K(3.1) | H51P(2.1) |  |  |  |  |
| Ko *et al.* (2019) (24) | **S450L(25.6)** | **S315T(12.8)** | I21T(2.6) | **M306V(7.7)** | S18Ter(2.6) | A90V(5.1) | **K43R(7.7)** |  |  |
|  | **H445Y(2.6)** | S315N(2.6) | I25T(2.6) | **M306I(5.1)** | T47P(2.6) | **D94G(2.6)** | K88Q(2.6) |  |  |
|  | R552L(2.6) | L378R(2.6) | S94A(5.1) | M306L(2.6) | H82Pfs(2.6) | D94N(2.6) |  |  |  |
|  |  | Y597D(2.6) |  | Y319S(2.6) | L85R(2.6) |  |  |  |  |
|  |  |  |  | I419V(2.6) |  |  |  |  |  |
| Farhat *et al.* (2016) (8, 25) |  |  |  |  |  | A90V(10.8) |  |  |  |
|  |  |  |  |  |  | **D94G(13.3)** |  |  |  |
|  |  |  |  |  |  | D94Y(2.1) |  |  |  |
| Park *et al.* (2018) (26) | **S450L(63.3)** | **S315T(93.3)** | Y113F(3.3) | **M306V(16.7)** | A46E(6.7) | A90V(3.3) | **K43R(20.0)** |  |  |
|  |  | Y113F(3.3) |  | **M306I(20.0)** |  | S91P(3.3) | K88R(6.6) |  |  |
|  |  |  |  | Y319D(6.7) |  |  |  |  |  |
| Farhat *et al.* (2016) (27) | **S450L(OR 70.0)** | **S315T(OR 169.0)** | **C(-15)T(OR 18.5)** | **M306V(OR 14.2)** | H51R(OR inf) | **D94G(OR 228.1)** | **K43R(OR 25.5)** | **A1401G(OR 127.4)** | **A1401G(OR 222.9)** |
|  | **D435V(OR 28.0)** |  |  | **M306I(OR 6.0)** |  | D94Y(OR inf) | K88R(OR inf) |  | A514C(OR 6.4) |
|  |  |  |  | Q497R(OR 9.5) |  | A90V(OR 126.8) |  |  |  |
|  |  |  |  |  |  | D94A(OR inf) |  |  |  |

Targets selected for primer-probe set design are indicated in bold.

INH, isoniazid; RIF, rifampin; EMB, ethambutol; PZA, pyrazinamide; FQ, fluoroquinolone; SM, streptomycin; KM, kanamycin; CAP, capreomycin; AMK, amikacin; OR, odds ratio

Supplementary Table 2. Description of the primers and probes used in this study

| Panel |  | Primer/probe | Sequence (5’->3’) | Concentration (nmol/L) | Dye |
| --- | --- | --- | --- | --- | --- |
| 1 |  | *IS6110*-F | GGCGTACTCGACCTGAAAGA | 450 |  |
|  |  | *IS6110*-R | CTGAACCGGATCGATGTGTA | 450 |  |
|  |  | *IS6110* | ACCATACGGATAGGGGA | 125 | VIC |
|  |  | *rpoB*-F | AGGAGTTCTTCGGCACCAG | 900 |  |
|  |  | *rpoB*-R | AGCCGATCAGACCGATGTT | 900 |  |
|  |  | *rpoB*450 | CCGACTGTTGGCGC | 250 | FAM |
|  |  | *rpoB*445 | TTGACCTACAAGCGCCGA | 250 | FAM |
|  |  | *rpoB*435 | AATTCATGGTCCAGAACA | 250 | FAM |
|  |  | *inhA*-F | GCTCGTGGACATACCGATTT | 1800 |  |
|  |  | *inhA*-R | CTTCAGTGGCTGTGGCAGT | 1800 |  |
|  |  | *inhA*(-15) | GGCGAGATGATAGGT | 500 | FAM |
|  |  | *katG*-F | GGGCTGGAAGAGCTCGTAT | 900 |  |
|  |  | *katG*-R | CCGTACAGGATCTCGAGGAA | 900 |  |
|  |  | *katG*315 | ATCACCACCGGCATC | 250 | VIC |
| 2 |  | *embB*-F | GTCGGACGACGGCTACATC | 1800 |  |
|  |  | *embB*-R | GCGGAAATAGTTGGACATGTAG | 1800 |  |
|  |  | *embB*306(V) | CCTGGGCGTGGCCCGAGTC | 250 | HEX |
|  |  | *embB*306(I) | CCTGGGCATHGCCCGAGTCG | 500 | HEX |
|  |  | *rpsL*-F | GCAGCGTCGTGGTGTATG | 450 |  |
|  |  | *rpsL*-R | CCTCGACCTGACTCGTCAAC | 450 |  |
|  |  | *rpsL*43 | CTCCGAGGAAGCCG | 125 | FAM |
|  |  | *rrs*-F | GTAATCGCAGATCAGCAACG | 1800 |  |
|  |  | *rrs*-R | CTCCCTCCCGAGGGTTAG | 1800 |  |
|  |  | *rrs*(1401) | CCCGTCGCGTCAG | 500 | VIC |
|  |  | *gyrA*-F | AGACCATGGGCAACTACCAC | 450 |  |
|  |  | *gyrA*-R | GCTTCGGTGTACCTCATCG | 450 |  |
|  |  | *gyrA*94 | TCTACGGCASCCTGG | 125 | FAM |

Supplementary Table 3. Mutation statuses of DNA material of nine MTB strains with known mutation purchased from the Korean National Tuberculosis Association

| Sample No. | Susceptibility phenotype | *katG* | *inhA* | *rpoB* | *embB* | *pncA* | *gyrA* | *rrs* | *rpsL* |
| --- | --- | --- | --- | --- | --- | --- | --- | --- | --- |
| 16 | Pan-Susceptible | - | - | - | - | - | - | - | - |
| 18 | Pan-Susceptible | - | - | - | - | - | - | - | - |
| 9 | Multidrug-Resistant | S315T R463L | - | D435V | M306V | D136N | D94G | - | - |
| 46 | Multidrug-Resistant | S315T R463L | - | S450L | Q497K | 74 C → del | A90V | - | K43R |
| 107 | Multidrug-Resistant | S315T | -15 C → T | S450L | M306I G406D | L35P | - | - | - |
| 2 | Extensively drug-resistant | S315T R463L | - | D435V | - | C14W | - | 1401 A → G | K43R |
| 23 | Extensively drug-resistant | R463L | -15 C → T | S450L | M306I | P62L | D94G | 1401 A → G | - |
| 32 | Extensively drug-resistant | R463L | -15 C → T | S450L | M306L | G97S | A90V | 1401 A → G | - |
| 77 | Extensively drug-resistant | S315T R463L | - | S450L | M306V | - | D94G | 1401 A → G | - |

The term “Extensively drug-resistant” indicates these were resistant to INH, RIF, FQ, and at least one second line injectable drug, which was its definition when the strains were referred to the Korean National Tuberculosis Association

Supplementary table 4. Demographic characteristics of 18 MDR-TB samples obtained from 16 patients

| Variables | Patients/samples (n=16/18) |
| --- | --- |
| Age, years |  |
| Mean (SD) | 66.89/66.0 (14.6/14.9) |
| Sex |  |
| Male (%) | 10/11 (62.5/61.1) |
| Female (%) | 6/7 (37.5/38.9) |
| Sample type (per sample) |  |
| Sputum | 16 (88.9) |
| Bronchial washing | 2 (11.1) |
| Resistance phenotype (per patient) |  |
| INH | 8 (50.0) |
| RIF | 7 (43.8) |
| EMB | 5 (31.3) |
| PZA | 4 (25.0) |
| FQ | 1 (6.3) |
| SM | 3 (18.8) |
| KM | 0 (0.0) |
| CAP | 0 (0.0) |
| AMK | 0 (0.0) |
| Unknown* | 5 (31.3) |
| Culture (per sample) |  |
| Positive | 12 (66.67) |
| Negative | 4 (22.2) |
| Not done | 2 (11.1) |
| AFB smear (per sample) |  |
| 3+ | 1 (5.6) |
| 2+ | 3 (16.7) |
| 1+ | 1 (5.6) |
| Negative | 13 (72.2) |

SD, standard deviation; AFB, Acid-fast bacillus

*Undetermined resistance phenotypes were cases where either the culture was negative or contaminated or positive, but drug sensitivity tests could not be conducted because the bacteria’s culturability was too low.

Supplementary Table 5. Xpert MTB/RIF assay results and ddPCR results

| Sample No. | Sample type | AFB smear | Xpert | | Probe | ddPCR (copies/well) | |
| --- | --- | --- | --- | --- | --- | --- | --- |
|  |  |  | MTB | RIF |  | *IS6110* | *rpoB* |
| G-1 | Sputum | Negative | Negative | Negative | - | 32.41 | - |
| G-2 | Sputum | Negative | Negative | Negative | - | - | - |
| G-3 | Sputum | Negative | Positive | Positive | probe E | 45.29 | 2.21 |
| G-4 | Sputum | Negative | Positive | Negative | - | 1493.43 | - |
| G-5.1 | Bronchial lavage | 2+ | Positive | Negative | - | 63.65 | - |
| G-5.2 | Sputum | 2+ | Negative | Negative | - | 2.33 | - |
| G-6.1 | Sputum | Negative | NT | NT | NT | 946.90 | - |
| G-6.2 | Bronchial lavage | Negative | NT | NT | NT | 268.66 | - |
| G-7 | Sputum | Negative | NT | NT | NT | 160733.52 | - |
| S-1 | Sputum | Negative | Positive | Positive | probe E | 162.28 | 13.03 |
| S-2 | Sputum | 3+ | Positive | Positive | probe E | 11.00 | 2.20 |
| S-3 | Sputum | 1+ | Positive | Positive | probe E | 195.64 | 7.66 |
| S-4 | Sputum | 2+ | Positive | Positive | probe E | 124.50 | 10.89 |
| S-5 | Sputum | Negative | Positive | Positive | probe B | - | - |
| S-6 | Bronchial lavage | Negative | Positive | Positive | probe B | - | - |
| S-7 | Sputum | Negative | Positive | Positive | probe A | 9.93 | 1.14 |
| S-8 | Sputum | 2+ | Positive | Positive | probe B | 1302.16 | - |
| S-9 | Sputum | Negative | Positive | Positive | probe E | 6.23 | - |
| S-13 | Sputum | Negative | Positive | Positive | probe E | - | - |
| S-14 | Sputum | Negative | Positive | Positive | probe B | 11.38 | - |

NT, not tested; -, not detected
